# Perception of Psychiatry by Other Healthcare Professionals (Physicians at Casablanca University Hospital)

**DOI:** 10.1192/j.eurpsy.2026.11963

**Published:** 2026-07-31

**Authors:** M. Sabir, N. Attouche, A. Tanjaoui, M. Agoub

**Affiliations:** Psychiatry, University Center for Psychiatry, Casablanca, Morocco

## Abstract

**Introduction:**

Mental health is a crucial component of overall well-being, with psychiatric disorders contributing significantly to morbidity and complicating other medical conditions. Morocco faces a particularly worrying situation, marked by a shortage of psychiatrists and unequal access to care. Understanding the perception of psychiatry among physicians is essential to improving the specialty’s attractiveness and strengthening training programs.

**Objectives:**

To analyze attitudes toward psychiatry among non-psychiatrist physicians.

To identify factors influencing these attitudes.

To propose actions for improving psychiatry’s representation and integration.

**Methods:**

We conducted a cross-sectional, descriptive, and analytical study among 305 physicians at Ibn Rochd University Hospital, excluding psychiatrists. Participants included interns, residents, attending physicians, and faculty members. Data were collected through a self-administered questionnaire and the ATP-30 scale. Analyses were performed using SPSS v27. Variables with p < 0.20 in univariate analysis were included in a multivariate linear regression model, with significance set at p < 0.05.

**Results:**

Physicians expressed an overall favorable perception of psychiatry, with a mean ATP-30 score of 115.9 (95% positive attitudes).

**Positive aspects:** Strong recognition of psychiatric patients’ humanity (88%) and the legitimacy of mental illness compared with physical conditions (91%). Psychiatry is generally considered a respected discipline.

**Challenges:** Doubts persist regarding therapeutic effectiveness (59% skeptical about improvement), concerns about side effects (72%), and a limited attractiveness of the specialty (46% uninterested).

**Influencing factors:** Gender, marital status, and professional status were significantly associated with attitudes, with women showing more favorable perceptions.

**Conclusions:**

Physicians at CHU Ibn Rochd showed generally positive attitudes toward psychiatry, consistent with international findings, but therapeutic pessimism remains a major concern. Addressing these reservations is crucial for promoting psychiatry, ensuring equitable access to mental healthcare, and better integrating it into the medical system.

**Disclosure of Interest:**

None Declared

